# Letter to the Editor

**DOI:** 10.22037/iej.2017.52

**Published:** 2017

**Authors:** SaravanaKarthikeyan Balasubramanian, Nidambur Vasudev Ballal

**Affiliations:** a Department of Conservative Dentistry and Endodontics, Srm Dental College, Srm University, Ramapuram, Chennai, Tamilnadu, India, ***E-mail:*** skmdc2006@gmail.com; b Department of Conservative Dentistry and Endodontics, Manipal College of Dental Sciences, Manipal University, Manipal, Karnataka, India.

**Sadr S** **,** **Golmoradizadeh A, Raoof M,** **Tabanfar MJ. Microleakage of Single-Cone Gutta-Percha Obturation Technique in Combination with Different Types of Sealers. Iran Endod J.** **2015 Summer;10(3):199-203. doi: 10.7508/iej.2015.03.011.**

Apical leakage continues to be a topic of great interest, due to the fact that clinical failures still occur, despite numerous advances in Endodontics. Most failures are probably attributed to the proliferation of bacteria that remain viable after chemo-mechanical preparation and cause peri-radicular infection [1]. I read with great interest, the article entitled “Microleakage of Single-Cone Gutta-Percha Obturation Technique in Combination with Different Types of Sealers” by Saeedeh Sadr *et al*. which has been published in your esteemed journal (IEJ Iranian Endodontic Journal 2015;10(3): 199-203). I want to share few of my thoughts regarding this study. It was a good study comparing the sealing ability of three root canal sealers including AH-26, glass ionomer cement (GIC) and zinc-oxide eugenol (ZOE) in single gutta-percha obturating system using the dye penetration method, but the authors can further redefine the study by incorporating few parameters. First, since the validity of dye leakage studies has been questioned because of the possible effect of entrapped air on ingress of the dye solution [2], dye leakage studies can be conducted under vacuum pressure. Studies have reported that vacuum pressure decreases the volume of entrapped air and allows complete dye penetration [3]. 

Second, though methylene blue has a high degree of staining and a molecular weight even lower than that of bacterial toxins, it possess disadvantages such as dissolution during the de mineralization and clearing process in addition to being difficult to observe the maximum penetration point in some cases [4]. On the other hand, Rhodamine-B dye presents greater diffusion in human dentin than methylene blue [5]. The molecules of Rhodamine-B dye are nanometric and are optimal to simulate bacterial enzymes and their toxins to assess microleakage. Other factors favouring the use of Rhodamine-B dye in leakage studies include: small particle size, water solubility, ease of visualization, better diffusability into dentinal tubules and hard tissue non-reactivity [6]. Hence, authors can attempt further similar studies using the above mentioned parameters for the better appreciation of the results.
