# Supplementary material for: Letter to the Editor
Source: Iran Endod J. 2017 Spring;12(2):266–7. doi: 10.22037/iej.2017.52 (PMC5421268; doi:10.22037/iej.2017.52)
Supplement: Related texts

# Authors Reply

**Published:** 2017

**Authors:** Maryam Raoof

**Affiliations:** Department of Endodontics, Dental School, Kerman University of Medical Sciences, Kerman, Iran, ***E-mail*:** maryam.raoof@gmail.com

We are grateful to the author for his/her interest and insightful comments on our manuscript entitled: Microleakage of Single-Cone Gutta-Percha Obturation Technique in Combination with Different Types of Sealers. We have attempted to answer his/her queries as much as possible. 

***Response for the first comment***

Although dye penetration tests are easy and cost-effective methods to evaluate the sealing ability of various dental materials, a number of criticisms have also been raised in relation to these tests. Sometimes the results cannot be compared with other studies because of the different methodological approaches [1]. According to the study by Spångberg et al. [2], entrapped of air bubbles produces artifacts in passive immersion technique. They suggested that entrapped air should be evacuated from the void before dye introduction. However, the elimination of air may lead to an overestimate of the in vivo extent of microleakage [3]. In our study, we didn't apply vacuum before the dye was introduced. This procedure appears to be more similar to clinical reality. 

***Response for the second comment***

Various types of dyes in different concentrations have been used in microleakage studies. Little attention, however, has been paid to the molecule sizes and specific behavior of materials in different circumstances [4]. Having high molecular weight and little capacity for ionic dissociation are the characteristics of both methylene blue and Rhodamine-B [5]. The molecule of Rhodamine-B is smaller than that of methylene blue dye. It is also less tensoactive than the latter and its penetration is deeper [5, 6]. Some studies also detected that Rhodamine-B showed the highest intradentinal penetration indexes and can be adequately visualized [6, 7]. Moreover, as a result of acidic pH and a possible reaction with dentin, methylene blue would be metabolized to colorless leukomethylene blue which is difficult to be visualized [8]. On the other hand, some authors reported that methylene blue favored the assessment of marginal microleakage compared to fluorescein and Rhodamine-B [9]. 

Despite the various advantages of Rhodamine-B, it has been used in a few studies when compared with other tracers. Many studies used methylene blue as dye since methylene blue is inexpensive, easy to manipulate and presents a high degree of staining as well as a molecular weight even lower than that of bacterial toxins [10]. However, there are some disadvantages with the use of methylene blue as a tracer in leakage studies. Dissolution during the demineralization and clearing process, in addition to being difficult to observe its maximum penetration point in some cases are some negative points [11]. Hence, we hold the view that further similar studies using the above mentioned parameters for the better appreciation of the results should be done.

## References

[1] Raskin A, D’Hoore W, Gonthier S, Degrange M, Déjou J (2001). Reliability of in vitro microleakage tests: a literature review. J Adhes Dent.

[2] Spangberg LSW, Acierno TG, Cha, BY (1989). Influence of entrapped air on the accuracy of leakage studies using dye penetration methods. J Endod.

[3] Beckham BM, Anderson RW, Morris CF (1993). An evaluation of three materials as barriers to coronal microleakage in endodontically treated teeth. J Endod.

[4] Taylor MJ, Lynch E (1992). Microleakage. Review J Dent.

[5] Heleno JF, Nunes E, Côrtes MI, Silveira FF (2012 Apr). Assessment of coronary microleakage marker capacity of three dyes. Dent Press Endod.

[6] Grempel M, Antoniazzi JH, Paiva JG (1990). Determinação da permeabilidade dentinária radicular natural de dentes humanos anteriores, segundo a faixa etária e o tipo de corante utilizado. Rev Paul Odontol.

[7] Hamaoka L, Moura AA (1996). Avalia.. o in vitro da permeabilidade dentin ria radicular, tendo como fonte de varia.. o trs diferentes tipos de corantes. Rev Odontol Univ So Paulo.

[8] Starkey DL, Anderson RW, Pashley DH (1993). An evaluation of the effect of methylene blue dye pH on apical leakage. J Endod.

[9] Diep EK, Berbert A, Bramante CM (1982). Infiltra.. o marginal em restaura es provis rias. RBO.

[10] Verissimo MD, Vale do SM (2006). Methodologies for assessment of apical and coronal leakage of endodontic filling materials:a critical review. J Oral Sci.

[11] Schafer E, Olthoff G (2002). Effect of three different sealers on the sealing ability of both thermafil obturators and cold laterally compacted Gutta- Percha. J Endod.
